# Accelerating large-scale protein structure alignments with graphics processing units

**DOI:** 10.1186/1756-0500-5-116

**Published:** 2012-02-22

**Authors:** Bin Pang, Nan Zhao, Michela Becchi, Dmitry Korkin, Chi-Ren Shyu

**Affiliations:** 1 Informatics Institute, University of Missouri, Columbia, MO, USA; 2 Department of Electrical and Computer Engineering, University of, Columbia, MO, USA; 3 Department of Computer Science, University of Missouri, Columbia, 65211, MO, USA

## Abstract

**Background:**

Large-scale protein structure alignment, an indispensable tool to structural bioinformatics, poses a tremendous challenge on computational resources. To ensure structure alignment accuracy and efficiency, efforts have been made to parallelize traditional alignment algorithms in grid environments. However, these solutions are costly and of limited accessibility. Others trade alignment quality for speedup by using high-level characteristics of structure fragments for structure comparisons.

**Findings:**

We present *ppsAlign*, a parallel protein structure Alignment framework designed and optimized to exploit the parallelism of Graphics Processing Units (GPUs). As a general-purpose GPU platform, *ppsAlign *could take many concurrent methods, such as TM-align and Fr-TM-align, into the parallelized algorithm design. We evaluated *ppsAlign *on an NVIDIA Tesla C2050 GPU card, and compared it with existing software solutions running on an AMD dual-core CPU. We observed a 36-fold speedup over TM-align, a 65-fold speedup over Fr-TM-align, and a 40-fold speedup over MAMMOTH.

**Conclusions:**

*ppsAlign *is a high-performance protein structure alignment tool designed to tackle the computational complexity issues from protein structural data. The solution presented in this paper allows large-scale structure comparisons to be performed using massive parallel computing power of GPU.

## Background

Large-scale protein structure comparison is becoming a more and more important approach to providing a better picture for understanding biological systems [1,2]. Given a database of protein structures, the main goal is either to find proteins that are structurally similar to a given protein (i.e., one-against-all comparison) or to build various connectivity among proteins by performing exhaustive comparisons on the whole database (i.e., all-against-all comparison). The results of structural comparison are useful in discovering potential structural, evolutionary, and functional relationships among these proteins and have significant impact on structure-based drug design [3], protein-protein docking [4], and other biological findings [5]. Recently, the dramatic increase in protein structural data [6] has led to an ever increasing demand for structure alignment tools that can not only find accurate alignments at residue level but also complete large-scale structure comparisons in a reasonable time.

Several approaches have been developed to address the limitations of traditional alignment methods and tackle the computational issues. The traditional alignment methods [1,2,7], such as DALI [8], CE [9], TM-align [10], Fr-TM-align [11], and MAMMOTH [12], are based on the comparison of residues or fragments to build initial alignments which are optimized by various procedures, such as Monte-Carlo, combinational search, and dynamic programming. These methods can provide accurate alignments at the residue level but are usually computationally expensive, which makes them infeasible in coping with very large datasets. To accelerate this process, one approach is to map the protein structures into 1D sequences and then use various sequence alignment methods to align two structures [13,14]. Another approach [15] utilizes a "bag of words" method, which depends on frequency of specific structural patterns, to provide speedy structure match and filtering. These approaches significantly improve efficiency for large datasets; however, this is often achieved at the cost of loss of topological details, which could lead to lower accuracy than the traditional structural comparison methods or could be unsuitable to perform residue-level alignment. Another approach is to parallelize traditional algorithms using a cluster or grid environment consisting of thousands of computing nodes [16,17]. These approaches can fulfill the desires of efficiency and accuracy but require high-performance computing environments which are energy-consuming and may not be accessible to the biologists.

With the increase in performance and programmability of many-core Graphic Processing Units (GPUs), more and more bioinformatics applications have been deployed on GPUs and have shown promising results in terms of speedup over their conventional CPU implementations. Liu et al. [18] implemented a GPU-based Smith-Waterman algorithm [19] for pair-wise DNA sequence alignment. Later, the efficiency of sequence alignments has been continuously improved in [20-23]. Vouzis and Sahinidis developed GPU-BLAST (Basic Local Alignment Search Tool) [24] to accelerate NCBI-BLAST [25]. Hung et al. developed a method for calculating RMSD (Root Mean Square Deviation) after superposition for ATI GPU card [26]. Stivala et al. utilized simulated annealing (SA) to develop a protein substructure searching algorithm, SA Tableau Search, to find structural motif at level of secondary structure element (SSE) [27]. It is worth mentioning that from the literature the SA Tableau Search is the first attempt to apply GPU in protein structure comparison at the SSE level. Other applications include protein-protein docking [28] and statistical phylogenetics [29].

In this paper, we present *ppsAlign*, a *parallel protein structure Alignment *framework which is designed and optimized to exhaustively exploit the parallelism of the GPU architecture for residue-level structure comparisons. Our experimental results (reported on a NVIDIA Tesla C2050 GPU card) show that *ppsAlign *significantly outperforms existing structural alignment tools in computational efficiency.

We believe that GPU's massive parallel computing power can unlock the door to a cost-effective and high-performance computing environment that can be beneficial to the structural biology community.

## Findings

### Overview

**Figure 1:**
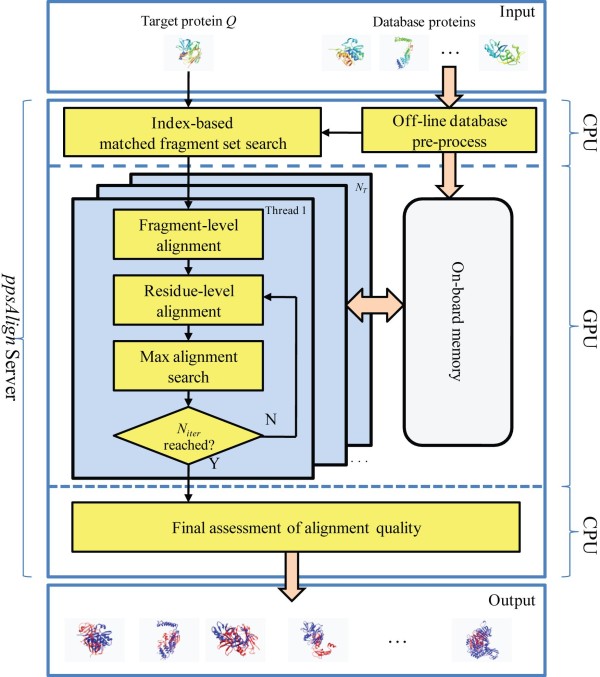
**Framework of *ppsAlign***. The framework consists of both GPU- and CPU- based processes. The input includes a target protein and database proteins. The output contains all the structural alignment results between the target protein and each database protein.

The framework of *ppsAlign *is shown in Figure 1. The inputs include a target protein and a protein database *Λ *= {*P*_1_, *P*_2_, ...., *P_n_*}. The outputs are structure alignments between the target protein and each database protein. The online alignment starts with a generation of some initial sets of matched fragments and corresponding alignments. Then, the initial alignments are extended and refined using Dynamic Programming to obtain the final results. Specifically, the *ppsAlign *algorithm consists of 5 steps: 1) Index-based matched fragment set (MFS) search is utilized to find the maximal *N_seed _*seed MFS' between the target protein and each database protein; 2) Fragment-level alignment is used to assemble the MFS' and generate initial alignments; 3) Residue-level alignment is used to refine the initial alignments to residue alignments; 4) Maximal alignment search is used to find a transformation that can best superimpose the entire target protein over each database protein based on the obtained residue alignments; 5) Final assessment is performed to calculate z-Score and evaluate statistical significance of alignments. Steps 1) and 5) are executed on the CPU core, while steps 2) ~ 4), the most time-consuming parts of *ppsAlign*, are implemented as GPU kernels and iteratively executed on GPU for *N_iter _*times. The GPU kernels are developed using CUDA (Compute Unified Device Architecture) programming model [30]. During the alignment, the protein structures and intermediate results from each GPU kernel are stored in GPU's on-board memory, such as read-only constant memory, read-only texture memory, and read-write global memory. Generally, the constant and texture memory have limited capacity but high access rate compared to the global memory. For an overview of GPU architecture and CUDA model, readers are referred to [30,31]. To facilitate the search of structurally similar fragments from the protein database, *ppsAlign *has an off-line component that pre-processes substructures from the entire protein database and builds an indexing tree to allow fast retrievals.

### Index-based matched fragment set search

The purpose of this CPU-based step is to quickly find all possible matched fragment sets (MFS') between the target protein and each database protein for further refinement based on an information retrieval (IR) approach which goes beyond the capability of the traditional "bag of words" concept by introducing spatial relationships among these fragments. Let $Q=\left\{q_{1,}q_{2},\ldots ,q_{L_{Q}}\right\}$ and $P=\left\{p_{1,}p_{2},...,\right)\left(p_{Lp}\right\}$ be a target protein with *L_Q _*residues and a database protein with *L_P _*residues, respectively. Here, *q *and *p *represent 3D coordinates of the *C_α _*atoms. A fragment *f *is a set of *L_f _*( = 8) continuous residues with the direction from *N *terminal to *C *terminal along the protein backbone. A MFS includes two non-empty subsets, *F_Q _*and *F_P_*, which contain an order of fragments that conforms to some criteria of structural similarity between *Q *and *P*, respectively. The fragments in a MFS will then be used to generate a rough alignment between *Q *and *P *in the fragment-level alignment.

The MFS search utilizes the substructure mapping method of the Index-based Substructure Alignment algorithm [32], developed by the authors, to retrieve similar fragments from the database proteins. In this method, substructures of the database proteins, extracted by a large set of pairs of windows along the backbones, are indexed off-line by an indexing tree in which similar substructures are clustered into same leaf node, denoted by $t_{i}^{\Lambda }$, and one substructure is selected as representative for each leaf node. Such representative structures preserve certain topological information, both locally and globally, from two disjoint substructures with various ranges of distances. Similarly, substructures in the target protein *Q *are indexed by an indexing tree in which each leaf node is denoted by $t_{i}^{Q}$. The representative substructure of each $t_{i}^{Q}$ is used to search the indexing tree of database and a list of best matched *t^Λ ^*is returned. For simplicity, we use *t *to denote $t_{i}^{Q}$ and *t^Λ^*. The database proteins that have substructures in *t^Λ ^*can be found by an inverted index. Such a database protein, *P*, can be represented by an order of substructures, denoted by *Ω_t_*, occurring in *t*. Likewise, the protein *Q *can be represented by an order of substructures, denoted by $\Omega_{t}^{Q}$, occurring in *t*. As substructures identified by the same *t *are similar, they can be used as "anchors" for rough alignments. For detailed explanation of the substructure mapping method, readers are referred to [32].

In *ppsAlign*, substructures are further projected into fragments as follows: if any residue of a substructure from $\Omega_{t}^{P}$ (or $\Omega_{t}^{Q}$) is located in a fragment, the fragment is selected and added to *F_P _*(or *F_Q_*). The fragment subsets *F_P _*and *F_Q _*are used to construct a MFS between the protein *Q *and *P*. After searching all *t^Q^*, we can obtain all possible MFS' between *Q *and database proteins, if any. In this step, if the algorithm cannot find any MFS for a database protein, all the fragments from *Q *and the database protein are selected to form a MFS. An example of MFS searching and construction is illustrated in Additional file 1: Figure S1.

After searching MFS, a filtering process is called to remove redundant MFS'. Then, the non-redundant MFS' between *Q *and each database protein are ranked according to scoring function *S_MFS _*and the top *N_seed _*sets are selected. The scoring function is defined as follows:

$${\text{S}}_{\text{MFS}}={\text{w}}_{\text{1}}\cdot \frac{{\text{N}}_{\text{Q}}}{{\text{N}}_{\text{f}}^{\text{Q}}}+{\text{w}}_{\text{2}}\cdot \frac{{\text{N}}_{\text{P}}}{{\text{N}}_{\text{f}}^{\text{P}}}+{\text{w}}_{\text{3}}\cdot \frac{\text{min}\left({\text{N}}_{\text{Q,}}{\text{N}}_{\text{P}}\right)}{\text{max}\left({\text{N}}_{\text{Q,}}{\text{N}}_{\text{P}}\right)}$$

where *N_Q _*and *N_P _*denote the cardinality of *F_Q _*and *F_P _*in a MFS, respectively.

$N_{f^{Q}}=\left\lceil L_{Q}/L_{f}\right\rceil \;\;and\;N_{f^{P}}=\left\lceil L_{P}/L_{f}\right\rceil$ are the numbers of fragments in the target protein and a database protein, respectively. The third term of the above scoring function is used to favor MFS' which have comparable *N_Q _*and *N_P_*. The values *w_1_, w_2_*, and *w_3 _*are used to weight the contributions from the three terms.

The data needed by *ppsAlign *in order to compute the alignments on GPU are: structures of the protein *Q *and of the database proteins, and MFS'. To allow efficient processing, those data must be judiciously laid out on the GPU memories. Specifically, the database structures are transferred to the texture memory before execution. The MFS' are transferred from CPU memory to GPU global memory as inputs to the fragment-level alignment (see Figure 1). Finally, the structure of protein *Q *is stored in the constant memory, which has smaller capacity but lower access latency compared to the texture memory.

### Fragment-level alignment

In this step, the fragments in each MFS are assembled to obtain initial alignments using Dynamic Programming (DP). For a given MFS, the DP algorithm first sorts the fragments from *F_Q _*and *F_P _*according to their locations in *Q *and *P*. Then, it computes the similarity score *S_f_*(*i, j*) of each fragment pair for 1 ≤ *i *≤ *N_Q _*and 1 ≤ *j *≤ *N_P _*using the following recurrence:

$$s_{f}\left(i,j\right)=\text{max}\left\{\begin{array}{c} S_{f}\left(i-1,j-1\right)+S_{f}\left(i,j\right)\\ S_{f}\left(i,j-1\right)+G_{f}\\ S_{f}\left(i-1,j\right)+G_{f} \end{array}\right),$$

where *G_f _*is gap penalty and *S_f _*is based on the inverse cosine distance of fragment's feature vector. Given a fragment pair, *A *and *B*, and their corresponding feature vectors *D_A _*and *D_B_, S_f _*is calculated as follows:

$$s_{f}=1-{\text{cos}}^{-1}\left(\frac{\left\langle D_{A},D_{B}\right\rangle }{∥D_{A}∥∙∥D_{B}∥}\right)$$

where <*D_A_, D_B _*> is the inner product of *D_A _*and *D_B_*, ||*D_A_*|| and ||*D_B_*|| are the norm of *D_A _*and *D_B_*, respectively. In the current implementation, features only use Euclidean distance of each residue pair for fast calculation. The main reason for using feature distance as an approximate measure of fragment similarity is the need for simple control paths due to the SIMT (Single Instruction, Multiple Thread) computing mode of the GPU [30]. Traditional methods usually calculate RMSD and find an optimal transformation using the Kabsh algorithm [33], which contains complex control flows and is therefore not suitable for the SIMT mode. This step provides a rough alignment result which will be refined by the residue-level alignment.

#### GPU computation for fragment-level alignment

**Figure 2:**
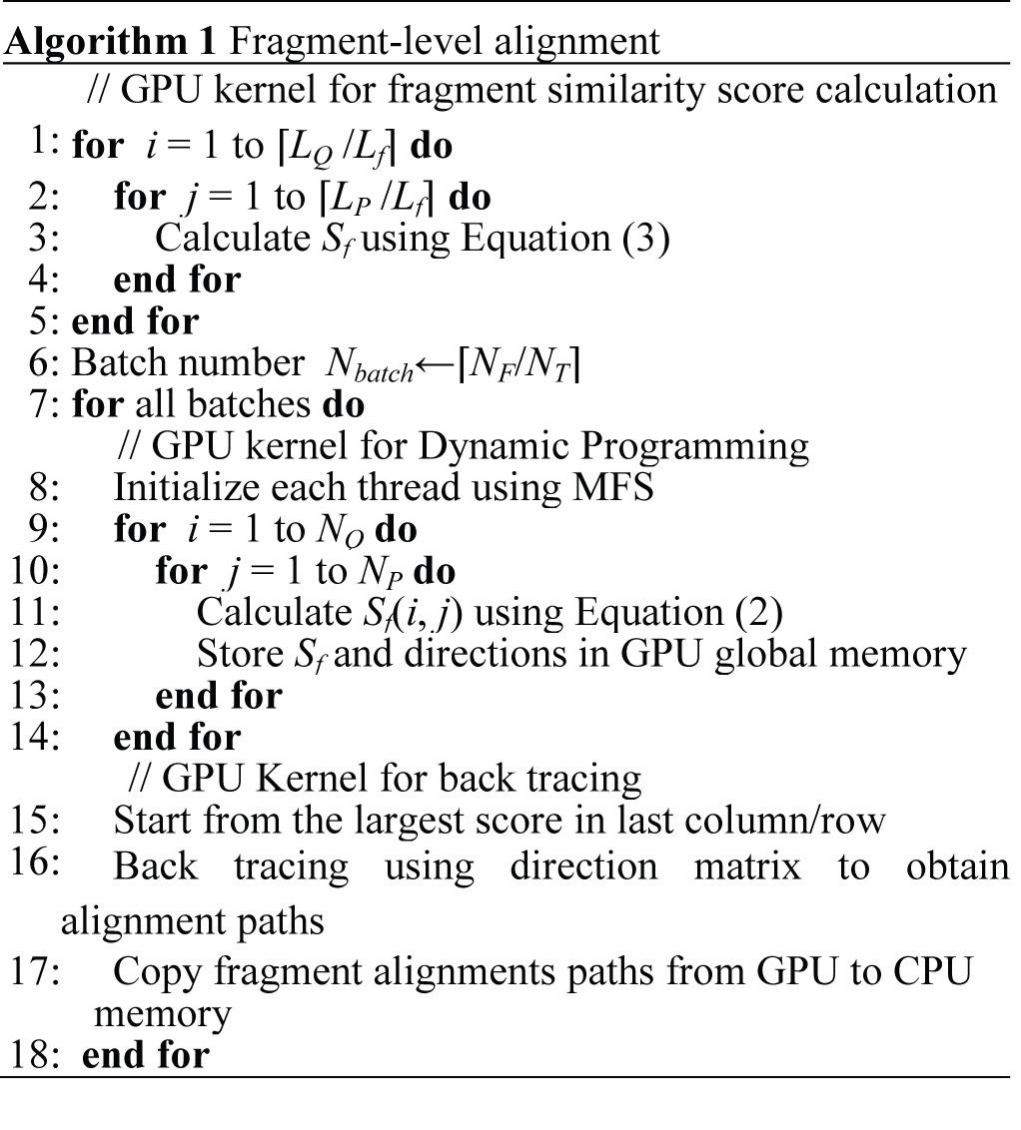
**Algorithm of fragment-level alignment**. Fragment-level alignment consists of three GPU kernels. The first kernel performs the computation of the fragment scores. The second kernel implements the Dynamic Programming algorithm, and the third one performs the back tracing.

The pseudo-code in Figure 2 describes the fragment-level alignment. The algorithm splits the computation into three GPU kernels. The first kernel performs the computation of the fragment scores *S_f _*by assigning a database protein to each thread. This kernel performs all-against-all fragment comparisons and writes similarity scores into the GPU global memory.

The second GPU kernel implements the DP algorithm, whereas the third one performs back tracing. The total number of threads *N_T _*that can run concurrently on the GPU is mainly limited by the global memory capacity of the GPU (in this phase each thread requires approximately 10 kB of memory). Suppose that the total number of MFS between *Q *and all database proteins is *N_F_*. If *N_F _*>*N_T_*, the overall MFS' will be divided into *N_batch _*= ⌈*N_F_*/*N_T_*⌉ batches. *ppsAlign *sequentially schedules each batch to run on GPU. In each batch, the DP is first executed as a GPU kernel and each thread corresponds to a MFS. Then, the GPU kernel for the back tracing is called to obtain alignment paths for each MFS. When a batch terminates, *ppsAlign *transfers the output (i.e., alignment path for each MFS) from the GPU memory to CPU memory. After aggregating the outputs from all batches, *ppsAlign *first performs filtering to remove redundant alignments, and then assembles all the fragments along the alignment paths to form residue alignments which will be further refined by the residue-level alignment.

**Figure 3:**
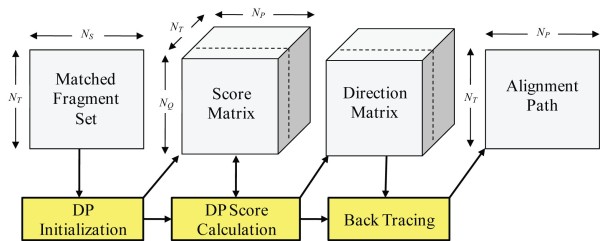
**GPU global memory layout of fragment-level alignment**. The Matched Fragment Sets (MFS') are stored in a 2D block. The score and direction matrices are stored in a separate 3D memory block. The alignment paths are stored in a 2D block.

It is critically important to effectively utilize the limited memory resources of the GPU. Our GPU memory allocation scheme is exemplified in Figure 3. The MFS' are stored in a 2D block of size (*N_T _*× *N_S_*) where *N_S _*is the maximal size of all MFS'. Each thread of the DP kernel fetches a MFS to initialize its setting. The score and direction matrices are stored in a separate 3D memory block of size (*N_Q _*× *N_P _*× *N_T_*), where *N_Q _*and *N_P _*represent the maximal number of fragments from the target protein and all the database proteins, respectively. The alignment paths are then stored in a 2D block of size (*N_P _*× *N_T_*). In *ppsAlign*, multiple GPU memory accesses are coalesced into a single transaction whenever possible. This fragment-level alignment process provides a selection of seed fragments which are likely to be successful in accurate alignment. Only approximately 1.6% of the total execution time is spent in this phase.

### Residue-level alignment

The results of fragment-level alignment are then refined by a residue-level alignment process. Such a refined alignment result is an ordered set *R *= {(*q_i_, p_i_*) | *q_i_*∊*Q', p_i_*∊*P'*}, where *Q' *⊆ *Q *(target protein) and *P' *⊆ *P *(database protein).

In this step, a rigid-body transformation (rotation and translation) *T *that minimizes the RMSD of *R *is first calculated. Then, the transformation *T *is used to superimpose all the residues from *Q *over *P*. Finally, the DP algorithm is used to find an alignment path between *Q *and *P *similar to the fragment-level alignment. In the DP, the gap penalty *G_r _*is set to 0 and the residue similarity score *S_r _*uses the scoring function from TM-align [10]. However, our framework can be configured to use any suitable residue-level scoring function [1].

As we mentioned previously, the complex control flows present in the traditional method for computing *T *(e.g., Kabsch algorithm [33]) make it unsuitable for the SIMT computing model of GPU. To address this issue, we implement and optimize a fast algorithm using quaternion-based characteristic polynomial (QCP) [34], gRMSD-QCP, to determine the transformation *T *on GPU. In the gRMSD-QCP kernel, coordinates of residues from two protein structures are first written into the GPU global memory and origin of coordinate is moved to the center of coordinates for each protein. Then, the inner-product of two coordinate matrices is calculated, which is used by QCP for RMSD calculation. The work flow of gRMSD-QCP is relatively simple, and therefore amenable of efficient GPU implementation.

#### GPU computation for residue-level alignment

The GPU implementation of residue-level alignment starts with loading coordinates of residues from *R *to the GPU global memory. Next, the gRMSD-QCP kernel is invoked to calculate the transformation *T *which is also written into the GPU global memory. Finally, a DP kernel is called to find residue alignments which are transferred into the CPU memory after the kernel terminates.

As in the fragment-level alignment phase, the residue-level alignments are divided into batches according to the memory requirement of the threads. After all the batches are executed, *ppsAlign *aggregates the outputs of residue alignment *R*, which are used in the next step for searching the maximal alignment.

### Maximal alignment search

The maximal alignment search is used to find the largest subset *M *⊆ *R *such that the score of the residue alignment *R*, denoted by *S_a_*, is maximized. Because finding the largest subset *M *is extremely time-consuming, a heuristic and approximate algorithm, MaxSub [35], has been developed to solve this problem. In *ppsAlign*, a variant of MaxSub, gMaxSub, is designed to parallelize the search process on the GPU. In the current implementation of *ppsAlign, S_a _*is defined using the TM-score [10].

#### GPU computation for maximal alignment search

**Figure 4:**
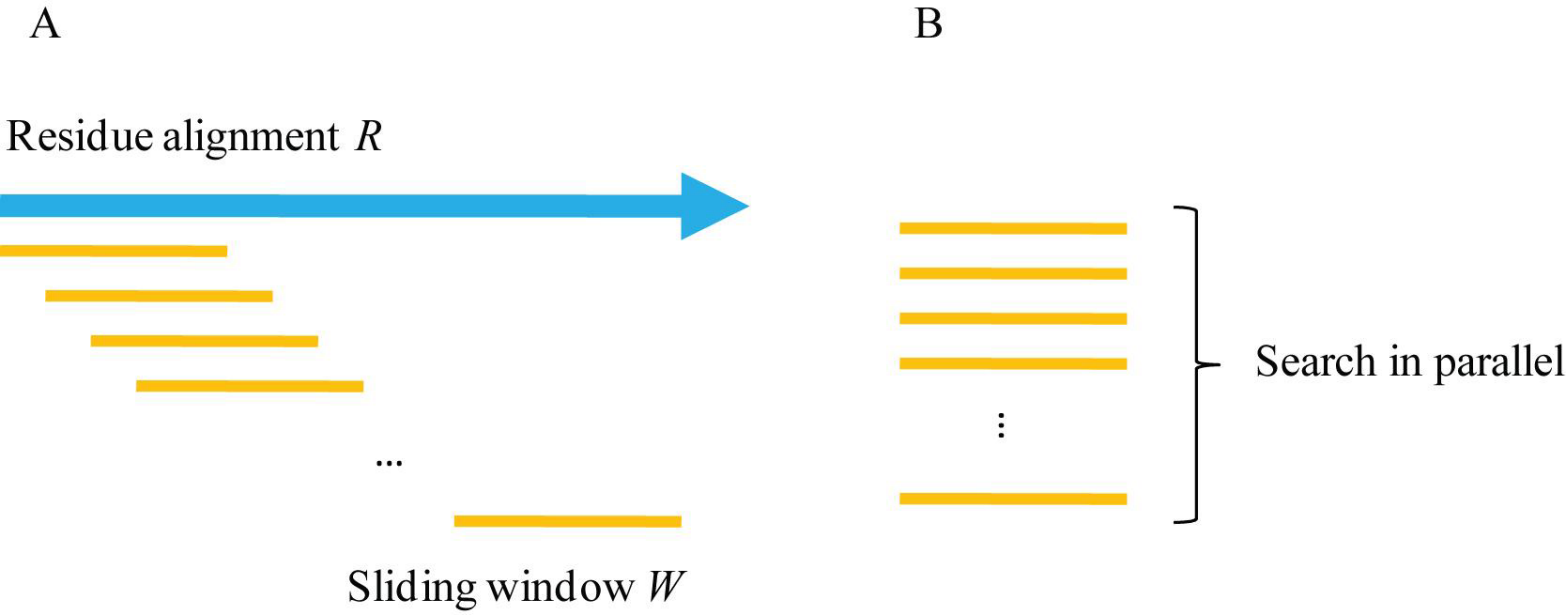
**Comparison of MaxSub and gMaxSub**. The original MaxSub algorithm on CPU searches the largest subset by shifting a window *W *along the residue alignment *R*. The gMaxSub searches the maximal alignment by concurrently dispatching each calculation of *W *to different GPU threads.

The input of this step is the alignment *R *from the residue-level alignment which has *L_R _*aligned residue pairs. The original MaxSub algorithm on CPU searches the largest subset *M *by shifting a window *W *of size *L_W _*along *R *(see Figure 4a). This results into (*L_R_*-*L_W _*+ 1) shift operations which are candidates for parallelization. Then, gMaxSub searches the maximal alignment by concurrently dispatching each calculation of *W *to different GPU threads (see Figure 4b).

**Figure 5:**
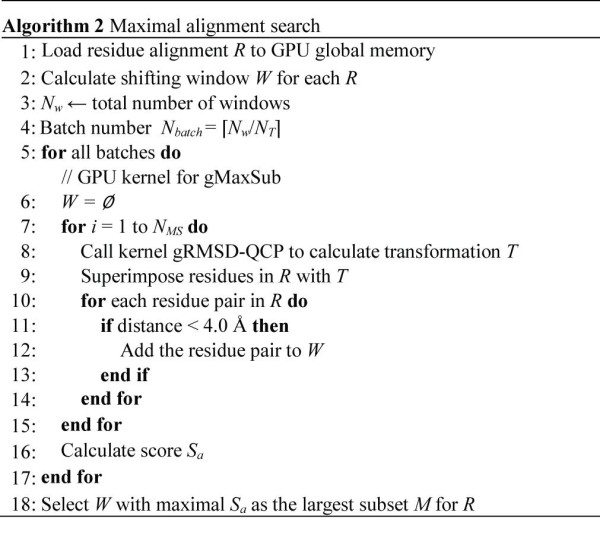
**Algorithm of maximal alignment search**. For each search, the gRMSD-QCP kernel is invoked to calculate the transformation superimpose residues with each window. The residue pair is added into the window if its distance is below a cutoff.

Figure 5 describes a pseudo-code of gMaxSub. First, for each residue alignment *R *between *Q *and *P*, (*L_R_*-*L_W _*+ 1) windows are generated. Second, the gRMSD-QCP kernel is invoked to calculate the transformation *T *for the residue pairs within each *W *and then *T *is used to superimpose residues from *Q *over *P *in *R*. Third, residue pair (*q_i_, p_i_*)∊*R *is added into *W *if its distance is below a cutoff (4.0 Å) after the superimposition. The above two steps (i.e., gRMSD-QCP and window extension) are iteratively executed for *N_MS _*times. Forth, the last *W *is assigned to *M *and *S_a _*is calculated.

As in previous phases, the maximal alignment searches are divided into batches. After all the batches are executed, *ppsAlign *aggregates the outputs of subset and selects the one with the largest *S_a _*as the largest subset *M*. The transformation *T *associated with the largest subset *M *is used to superimpose all the residues from *Q *over *P *and the residue pair whose distance is below a cutoff (4.0Ǻ) is selected to form a new residue alignment *R*.

After gMaxSub terminates, if the current iteration number <*N_iter_*, the residue alignment *R *will be first filtered to remove redundant alignments from the same database protein and then sent to the residue-level alignment for further refinement; otherwise, *R *will be used as input for the next step of final assessment.

### Final assessment of alignment quality

After structure alignments are computed on GPU, the residue alignments *R *are transferred from the GPU memory to CPU memory. We use PSI (percentage of structural similarity), defined as the percentage of residue pairs from *R *with distance below 4.0 Å, to score the alignment quality. We also assess the statistical significance of the alignments through z-Score of the PSI, which is given as follows:

$$\text{z - Score = }\frac{\text{PSI - }{\text{μ}}_{\text{PSI}}}{\sigma_{\text{PSI}}}$$

where *μ_PSI _*and *σ_PSI _*denote mean and standard deviation of PSI for a given protein chain length, respectively. The parameters *μ_PSI _*and *σ_PSI _*are obtained using a method similar to [12], leading to the following settings: *μ_PSI _*= 375.64·*k*^-0.5295 ^and *σ_PSI _*= 99.67·*k*^-0.5885^. Here, *k *is the minimum chain length between target and database proteins.

## Results

In this section, we compare *ppsAlign*'s performance to concurrent methods in terms of alignment quality and computational efficiency. We evaluate *ppsAlign *using an NVIDIA Tesla C2050 GPU card equipped with 448 cores at 1.15 GHz and 3 GB global memory. The concurrent methods include TM-align [10], Fr-TM-align [11], and MAMMOTH [12], which share similar computational framework as *ppsAlign*. As DALI [8] and CE [9] have been exhaustively evaluated elsewhere [10], we do not include these approaches in our experiments. We download software packages of these methods from their official websites and evaluate the performance on a Linux personal computer with AMD Opetron dual-core 1000 series processor at 1.8 GHz and 8 GB RAM.

The main purpose of structure alignment is to maximize the number of aligned residues (*N_e_*) while minimizing the RMSD of the aligned residues, denoted by cRMSD. To eliminate the size dependence of cRMSD on *N_e_*, in this paper we use a normalized measure of cRMSD, RMSD_100_, to evaluate the alignment quality. RMSD_100 _is calculated as follows [36]:

$$RSMD_{100}=\frac{cRMSD}{1+\;\text{ln}\sqrt{\frac{N_{e}}{100}}},$$

which corresponds to the cRMSD value expected if the two protein structures were 100 residues long.

To evaluate efficiency, we measure the execution time on a dataset in which the protein's chain length is in a range from 80 to 500 residues extracted from ASTRAL 1.75 database [37] with sequence identity < 40% (ASREAL40). The database protein chain length is determined by the global memory capacity on the GPU card. However, this limitation is not severe as 98.5% ASTRAL40 protein chains have less than 500 residues. We expect that the advancement of GPU technology will solve this memory limitation issue in the near future so that the *ppsAlign *algorithm can handle protein chains longer than 500 residues. Currently we can handle structures larger than 500 residues in one of the following two ways: 1) by sending the alignment tasks to our CPU-based algorithm and 2) if resource allows, by using another GPU card to align the remaining 1.5% of large structures. Although the algorithm can also handle small protein chains below 80 residues (~16% of ASTRAL40), we do not use them for our testing because they have relatively simple topologies [38].

To efficiently utilize global memory of GPU card, the entire database proteins are sorted according to the chain length and then divided into two small datasets: 1) *D_1_*, which includes 6, 569 proteins in the range [80, 250) residues selected from ASTRAL40 according to the length distribution of proteins, and 2) *D_2_*, which includes 1, 912 proteins in the range [251, 500) residues. The target dataset includes 100 proteins which are randomly selected in the range [80, 250) from ASTRAL40. For each target protein, a one-against-all alignment is performed with all database proteins and totally 100 × (6, 569 + 1, 912) = 848, 100 non-homologous protein pairs are compared during the experiment.

### Scalability of *ppsAlign*

**Figure 6:**
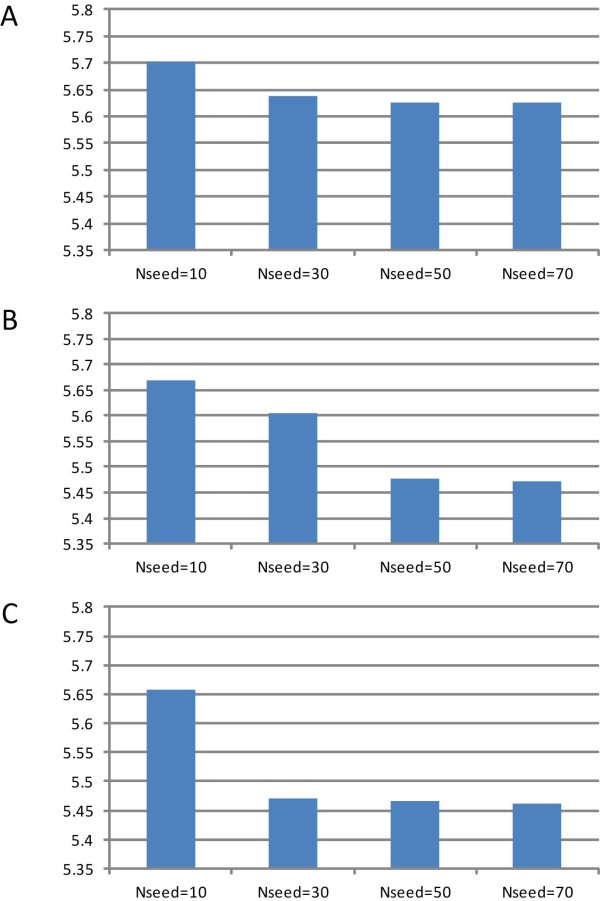
**Performance comparison of *ppsAlign *with different settings of *N_seed _*and *N_iter_***. *ppsAlign *is running on NVIDIA Tesla C2050 GPU card with a small target dataset of 17 proteins. The parameter settings of *ppsAlign *are *N_iter _*= {3, 5, 7} and *N_seed _*= {10, 30, 50, 70}. (**A**) *N_iter _*= 3. (B) *N_iter _*= 5. (C) *N_iter _*= 7.

There are two critical parameters for *ppsAlign*, namely the maximal number of iteration (*N_iter_*) and the maximal number of MFS (*N_seed_*). Intuitively, when increasing *N_iter _*or *N_seed_, ppsAlign *will often obtain better alignment quality but the execution time will be significantly lengthened. To verify this, we preliminarily investigate the performance of different settings using a small target dataset of 17 proteins and the dataset *D_1 _*in terms of RMSD_100_. The experimental results of RMSD_100 _with *N_iter _*= {3, 5, 7} and *N_seed _*= {10, 30, 50, 70} are shown in Figure 6, which illustrates that *ppsAlign *has decreased RMSD_100 _when *N_iter _*and/or *N_seed _*is increasing. This figure can be used as a guideline for parameter selection of *ppsAlign*. For a fair comparison of efficiency improvement from *ppsAlign *to a concurrent method, we select a combination of *N_iter _*and *N_seed _*that achieves comparable alignment quality.

### Speedup over TM-align and CPU-based *ppsAlign*

In this experiment, *ppsAlign *is executed with a parameter setting of *N_iter _*= 3 and *N_seed _*= 20 which results in a comparable RMSD_100 _to TM-align and the CPU version of *ppsAlign*. Table 1 summarizes the alignment quality, average execution time, and corresponding speedup. *ppsAlign *achieves speedups of 23.8 and 35.9 compared to CPU-based *ppsAlign *and TM-align, respectively. The detailed comparison of alignment quality of *ppsAlign *and TM-align can be found in Additional file 1: Table S1.

**Table 1:** Average execution time of TM-align, CPU-based *ppsAlign*, and *ppsAlign *with parameter settings (*N_iter _*= 3 and *N_seed _*= 20).

Dataset	Methods	RMSD_100_	Execution time (s)	Speedup of *ppsAlign*
*D_1_*	*ppsAlign*	5.7	64	-

	CPU-based *ppsAlign*	5.7	1596	24.9

	TM-Align	5.7	2170	33.9

*D_2_*	*ppsAlign*	5.3	41	-

	CPU-based *ppsAlign*	5.3	899	21.9

	TM-Align	5.3	1597	39.0

Total	*ppsAlign*		105	-

	CPU-based *ppsAlign*		2495	23.8

	TM-Align		3767	35.9

RMSD_100 _is the expected value of cRMSD if the two protein structures were 100 residues long. *ppsAlign *is running on NVIDIA Tesla C2050 GPU card and other methods (CPU-based *ppsAlign *and TM-align) are running on a computer with AMD Opetron dual-core 1000 series processor at 1.8 GHz and 8 GB RAM. The parameter settings of *ppsAlign *are *N_iter _*= 3 and *N_seed _*= 20

### Speedup over Fr-TM-align

Since Fr-TM-align performs more iterations to improve its alignment quality over TM-align, we increase both iteration and seed numbers of *ppsAlign *algorithm to achieve a comparable alignment quality with Fr-TM-align. The experimental results of RMSD_100_, average execution time, and corresponding speedup with *N_iter _*= 6 and *N_seed _*= 30 are shown in Table 2. *ppsAlign *achieves speedup 64.7 compared to Fr-TM-align with the same alignment quality. The detailed comparison of alignment quality of *ppsAlign *and Fr-TM-align can be found in Additional file 1: Table S2.

**Table 2:** Average execution time of Fr-TM-align and *ppsAlign *with parameter settings (*N_iter _*= 6 and *N_seed _*= 30).

Dataset	Methods	RMSD_100_	Execution time (s)	Speedup of *ppsAlign*
*D_1_*	*ppsAlign*	5.4	326	-

	Fr-TM-align	5.4	19849	60.9

*D_2_*	*ppsAlign*	5.1	224	-

	Fr-TM-align	5.1	15729	70.2

Total	*ppsAlign*		550	-

	Fr-TM-align		35578	64.7

RMSD_100 _is the expected value of cRMSD if the two protein structures were 100 residues long. *ppsAlign *is running on NVIDIA Tesla C2050 GPU card and Fr-TM-align is running on a computer with AMD Opetron dual-core 1000 series processor at 1.8 GHz and 8 GB RAM. The parameter settings of *ppsAlign *are *N_iter _*= 6 and *N_seed _*= 30

### Speedup over MAMMOTH

In the last experiment, we use the same dataset to compare the performance of *ppsAlign *and MAMMOTH. Different from TM-align and Fr-TM-align, MAMMOTH is originally developed for the purpose of large-scale comparisons with high efficiency at the cost of the reduction of alignment quality. Because of its high speed, MAMMOTH is used as a benchmark for maximal speed on the CPU platform in [39]. The experimental results of RMSD_100_, average execution time, and corresponding speedup with *N_iter _*= 1 and *N_seed _*= 8 are shown in Table 3. *ppsAlign *achieves speedup 40.3 compared to MAMMOTH and higher alignment quality. The detailed comparison of alignment quality of *ppsAlign *and MAMMOTH can be found in Additional file 1: Table S3.

**Table 3:** Average execution time of MAMMOTH and *ppsAlign *with parameter settings (*N_iter _*= 1 and *N_seed _*= 8).

Dataset	Methods	RMSD_100_	Execution time (s)	Speedup of *ppsAlign*
*D_1_*	*ppsAlign*	6.3	10	-

	MAMMOTH	10.3	470	47.0

*D_2_*	*ppsAlign*	5.9	8	-

	MAMMOTH	9.2	255	31.9

Total	*ppsAlign*		18	-

	MAMMOTH		725	40.3

RMSD_100 _is the expected value of cRMSD if the two protein structures were 100 residues long. *ppsAlign *is running on NVIDIA Tesla C2050 GPU card and MAMMOTH is running on a computer with AMD Opetron dual-core 1000 series processor at 1.8 GHz and 8 GB RAM. The parameter settings of *ppsAlign *are *N_iter _*= 1 and *N_seed _*= 8

## Discussion

The framework of *ppsAlign *is a general-purpose GPU platform for protein structure alignment which could take many concurrent methods, such as TM-align [10] and Fr-TM-align [11], into the parallelized algorithm design. An important novelty in our approach is to create a unique design to manage resources of the GPU architecture. First, an intelligent decomposition of the application in kernels characterized by different parallelization strategies is provided. In the existing methods for GPU-based sequence alignment mentioned previously, a pair-wise comparison is either assigned to a thread (i.e., inter-task parallelization) or corporately performed by a block of threads (i.e., intra-task parallelization) [18,20]. However, as the workflow of structure alignment is more complicated than that of sequence alignment, neither the inter- nor the intra- task parallelization can efficiently exploit the GPU computing power. Therefore, *ppsAlign *utilizes a hybrid inter- and intra- task parallel model. In particular, each task (i.e., pair-wise structural comparison) is divided into several independent seed alignments. Each seed alignment is assigned to a different thread (inter-task parallelization), whereas each block executes one or more pair-wise comparisons (intra-task parallelization). Second, a smart design of memory layout and memory access patterns are developed, the former allowing an effective use of the memory capacity at the different levels of the GPU memory hierarchy, and the latter minimizing the memory bandwidth requirement of the application. Third, several efficient algorithms for avoiding complex control flow on GPU are proposed to take advantage of the SIMT nature of the GPU. For instance, a feature-based measure is used to compute similarity of fragment at the fragment-level alignment which can avoid time-consuming RMSD calculation at the initial stage of structure alignment.

One of the major ways in which *ppsAlign *differs to other methods is implementing protein structure alignment *at the residue level *on GPU. Recently, the GPU-enhanced algorithms are gaining an increasing attention in bioinformatics. One of the major steps was a GPU implementation of a one-against-all sequence comparison using Smith-Waterman algorithm [20,21]. With these methods, a sequence database search can be performed resulting in a list of similarity scores, while these methods do not provide the detailed alignment information of the best hits [23]. To provide detailed residue-residue correspondence, GPU-BLAST [24] was developed, that allowed to accelerate the NCBI-BLAST search, achieving the speedup between 3 and 4 on an NVIDIA Tesla C2050 GPU card. In addition, another approach to protein sequence that uses backtracking on GPU to construct alignment of residues has been proposed [23]. Compared to the sequence alignments, the implementation of structure alignment on GPU is a more challenging task, because some routines (e.g., RMSD calculation) can cause severe divergence among GPU threads and decrease performance of GPU. One of the first structure comparison methods implemented on GPU, SA Tableau Search [27], aligns protein substructure at the secondary structure level, that is by aligning secondary structure elements, while not aligning structures at the residue level. To the best of our knowledge, *ppsAlign *is the first protein structure comparison platform for GPU that provides the residue level structural alignment.

The substantial contribution of *ppsAlign *is to provide a high-performance computing platform for the research community. An alternative solution to accelerate the protein structure alignment is to install more CPU computing cores in a single machine. However, using more CPU cores in a single machine need to upgrade main board and memory accordingly, which could decrease price/performance ratio. In contrast, installing a GPU card into a PCIe (Peripheral Component Interconnect Express) slot does not require extra cost and more GPU cards can be installed into one PCIe slot by a switch. In this paper, an NVIDIA Tesla C050 GPU card is utilized to evaluate performance, which has also been used in GPU-BLAST [24]. Though it is a high end product of NVIDIA, we expect its price will drop in the near future due to market demand in gaming industry.

## Conclusions

This paper presents *ppsAlign *for large-scale protein structure alignment using GPUs. *ppsAlign *employs an index-based search procedure to find seeds of matched fragment sets, and then iteratively refines the seeds with fragment- and residue- level alignments. We provide an in-depth comparison of *ppsAlign *against several concurrent CPU-based methods. Our experimental results show that *ppsAlign *can achieve significant speedup over its CPU implementation, TM-align, Fr-TM-align, and MAMMOTH on a single NVIDIA Tesla C2050 GPU.

We emphasize that the framework of *ppsAlign *is not designed as a replacement for the existing structural alignment tools, but as a general-purpose platform for protein structure alignments on GPU. With this platform, we can parallelize the existing algorithms (e.g., TM-align and Fr-TM-align) on GPU and utilize the massive parallel computing power of GPU to achieve high-throughput structural comparisons without sacrificing alignment quality.

## Availability and requirements

• Project name: *ppsAlign*

• Project home page: http://proteindbs.rnet.missouri.edu/ppsalign/ppsalign.html

• Operating system(s): Linux

• Programming language: CUDA, JAVA, and PHP

• License: none

## Abbreviations

BLAST: Basic Local Alignment Search Tool; CPU: Central Processing Unit; CUDA: Compute Unified Device Architecture; DP: Dynamic Programming; GPU: Graphics Processing Units; IR: information retrieval; MFS: matched fragment set; PCIe: Peripheral Component Interconnect Express; RMSD: Root mean square deviation; SA. simulated annealing; SIMT: Single Instruction: Multiple Thread; SSE: secondary structure element.

## Availability of supporting data

The data sets supporting the results of this article are available in the Worldwide Protein Data Bank repository, http://www.wwpdb.org/.

## Competing interests

The authors declare that they have no competing interests.

## Authors' contributions

BP developed the software and wrote the manuscript. NZ analyzed results and contributed to the discussion. MB and DK contributed to discussion, analyzed the results, and revised the manuscript. CRS coordinated the study and contributed to writing the manuscript. All authors read and approved the final manuscript.

## Supplementary Material

Additional file 1 **Figure S1**. In this example, one leaf node $t_{j}^{Q}$ from the indexing tree of the target protein *Q *is used to search the indexing tree of entire protein database *Λ *and *m *best matched nodes are returned. In this example, $t_{j}^{Q}$ node is represented by a representative $c_{j}^{Q}$ which is a "structure medium" from three similar substructures {*u_j, 1_, u_j, 2_, u_j, 3_*} from *Q*. A search of $c_{j}^{Q}$ on the indexing tree of *Λ *returns two database leaf nodes, $t_{i}^{\Lambda }$ and $t_{k}^{\Lambda }\cdot t_{i}^{\Lambda }$ node, represented by $c_{i}^{\Lambda }$, has two groups of similar substructures {*d_i, 1, 1_, d_i, 1, 2_*} and {*d_i, 2, 1_, d_i, 2, 2_, d_i, 2, 3_*} which are from database proteins *P_1 _*and *P_2_*, respectively. $t_{k}^{\Lambda }$ node, represented by $c_{k}^{\Lambda }$, has two groups of similar substructures {*d_k, 1, 1_, d_k, 1, 2_*} and {*d_k, 3, 1_, d_k, 3, 2_, d_k, 3, 3_*} from database proteins *P_1 _*and *P_3_*, respectively. The RMSD of $\left\{c_{j}^{Q},c_{i}^{\Lambda }\right\}$ and $\left\{c_{j}^{Q},c_{k}^{\Lambda }\right\}$ is below a cutoff (4.5Ǻ). After substructure searching, the target protein *Q *can be represented by $\Omega_{t}^{Q}$ = {*u_j, 1_, u_j, 2_, u_j, 3_*}. The database proteins *P_1_, P_2_*, and *P_3 _*can be represented by $\Omega_{t}^{P_{1}}$ = {*d_i, 1, 1_, d_i, 1, 2_, d_k, 1, 1_, d_k1, 2_*}, $\Omega_{t}^{P_{2}}$ = {*d_i, 2, 1_, d_i, 2, 2_, d_i, 2, 3_*}, and $\Omega_{t}^{P_{3}}$ = {*d_k, 3, 1_, d_k, 3, 2_, d_k, 3, 3_*}, respectively. After projecting the substructures to fragments, we have three MFS' for node *i *of the indexing tree of *Q *for *P_1_, P_2_*, and *P_3_*. Table S1. Comparison of alignment quality (RMSD_100_) of *ppsAlign *and TM-align. The table compares the alignment quality measured in RMSD_100 _of the 100 target proteins using *ppsAlign *and TM-align. Table S2. Comparison of alignment quality (RMSD_100_) of *ppsAlign *and Fr-TM-align. The table compares the alignment quality measured in RMSD_100 _of the 100 target proteins using *ppsAlign *and Fr-TM-align. Table S3. Comparison of alignment quality (RMSD_100_) of *ppsAlign *and MAMMOTH.The table compares the alignment quality measured in RMSD_100 _of the 100 target proteins using *ppsAlign *and MAMMOTH. Click here for file
