# Supplementary material for: Accelerating large-scale protein structure alignments with graphics processing units
Source: BMC Res Notes. 2012 Feb 22;5:116. doi: 10.1186/1756-0500-5-116 (PMC3309952; doi:10.1186/1756-0500-5-116)
Supplement: Additional file 1 — Figure S1. In this example, one leaf node tjQ from the indexing tree of the target protein Q is used to search the indexing tree of entire protein database Λ and m best matched nodes are returned. In this example, tjQ node is represented by a representative cjQ which is a "structure medium" from three similar substructures {uj, 1, uj, 2, uj, 3} from Q. A search of cjQ on the indexing tree of Λ returns two database leaf nodes, tiΛ and tkΛ⋅tiΛ node, represented by ciΛ, has two groups of similar substructures {di, 1, 1, di, 1, 2} and {di, 2, 1, di, 2, 2, di, 2, 3} which are from database proteins P1 and P2, respectively. tkΛ node, represented by ckΛ, has two groups of similar substructures {dk, 1, 1, dk, 1, 2} and {dk, 3, 1, dk, 3, 2, dk, 3, 3} from database proteins P1 and P3, respectively. The RMSD of cjQ,ciΛ and cjQ,ckΛ is below a cutoff (4.5Ǻ). After substructure searching, the target protein Q can be represented by ΩtQ = {uj, 1, uj, 2, uj, 3}. The database proteins P1, P2, and P3 can be represented by ΩtP1 = {di, 1, 1, di, 1, 2, dk, 1, 1, dk1, 2}, ΩtP2 = {di, 2, 1, di, 2, 2, di, 2, 3}, and ΩtP3 = {dk, 3, 1, dk, 3, 2, dk, 3, 3}, respectively. After projecting the substructures to fragments, we have three MFS' for node i of the indexing tree of Q for P1, P2, and P3. Table S1. Comparison of alignment quality (RMSD100) of ppsAlign and TM-align. The table compares the alignment quality measured in RMSD100 of the 100 target proteins using ppsAlign and TM-align. Table S2. Comparison of alignment quality (RMSD100) of ppsAlign and Fr-TM-align. The table compares the alignment quality measured in RMSD100 of the 100 target proteins using ppsAlign and Fr-TM-align. Table S3. Comparison of alignment quality (RMSD100) of ppsAlign and MAMMOTH.The table compares the alignment quality measured in RMSD100 of the 100 target proteins using ppsAlign and MAMMOTH. [file 1756-0500-5-116-S1.DOCX]

## Supplemental Materials

##
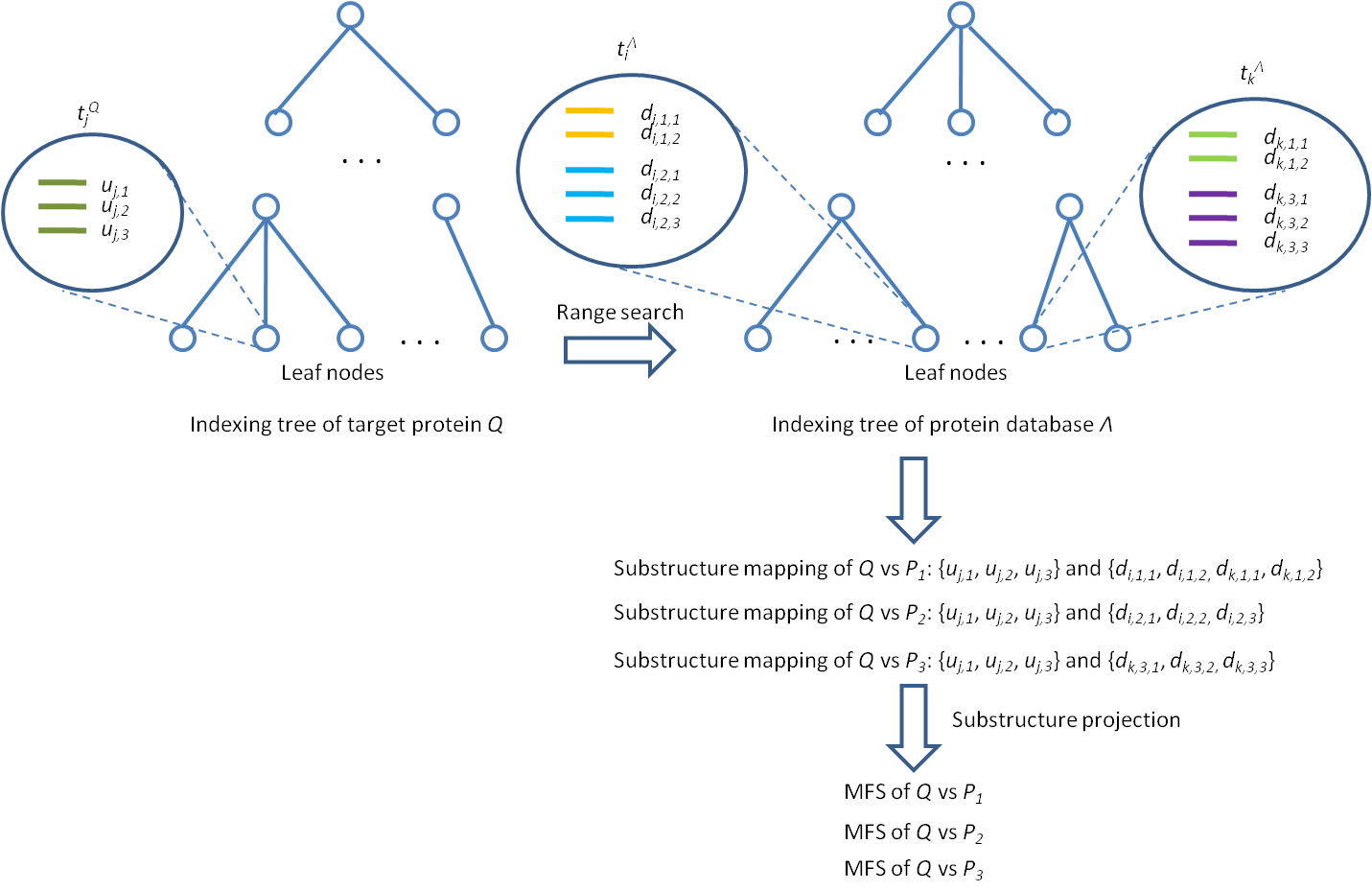


**Figure S1 - Example of index-based MFS search and construction.**

In this example, one leaf node *t_j_^Q^* from the indexing tree of the target protein *Q* is used to search the indexing tree of entire protein database *Λ* and *m* best matched nodes are returned. In this example, *t_j_^Q^* node is represented by a representative *c_j_^Q^* which is a “structure medium” from three similar substructures {*u_j,1_*, *u_j,2_*, *u_j,3_*} from *Q*. A search of *c_j_^Q^* on the indexing tree of *Λ* returns two database leaf nodes, *t_i_^Λ^* and *t_k_^Λ^*. *t_i_^Λ^* node, represented by *c_i_^Λ^*, has two groups of similar substructures {*d_i,1,1_*, *d_i,1,2_*} and {*d_i,2,1_*, *d_i,2,2_*, *d_i,2,3_*} which are from database proteins *P_1_* and *P_2_*, respectively. *t_k_^Λ^* node, represented by *c_k_^Λ^*, has two groups of similar substructures {*d_k,1,1_*, *d_k,1,2_*} and {*d_k,3,1_*, *d_k,3,2_*, *d_k,3,3_*} from database proteins *P_1_* and *P_3_*, respectively. The RMSD of {*c_j_^Q^*, *c_i_^Λ^*} and {*c_j_^Q^*, *c_k_^Λ^*} is below a cutoff (4.5Ǻ). After substructure searching, the target protein *Q* can be represented by *Ω_t_^Q^* ={*u_j,1_*, *u_j,2_*, *u_j,3_*}. The database proteins *P_1_*, *P_2_*, and *P_3_* can be represented by $\Omega_{t}^{P_{1}}$={*d_i,1,1_*, *d_i,1,2_*, *d_k,1,1_*, *d_k1,2_*}, $\Omega_{t}^{P_{2}}$={*d_i,2,1_*, *d_i,2,2_*, *d_i,2,3_*}, and $\Omega_{t}^{P_{3}}$={*d_k,3,1_*, *d_k,3,2_*, *d_k,3,3_*}, respectively. After projecting the substructures to fragments, we have three MFS’ for node *i* of the indexing tree of *Q* for *P_1_*, *P_2_*, and *P_3_*.

## Table S1 - Comparison of alignment quality (RMSD_100_) of *ppsAlign* and TM-align.

The table compares the alignment quality measured in RMSD_100_ of the 100 target proteins using *ppsAlign* and TM-align.

| SCOP ID | Dataset *D_1_* | | Dataset *D_2_* | | SCOP ID | Dataset *D_1_* | | Dataset *D_2_* | |
| --- | --- | --- | --- | --- | --- | --- | --- | --- | --- |
|  | *ppsAlign* | TM-align | *ppsAlign* | TM-align |  | *ppsAlign* | TM-align | *ppsAlign* | TM-align |
| d1f9ya_ | 5.5 | 5.4 | 5.2 | 5.2 | d1y6ha_ | 5.9 | 5.9 | 5.6 | 5.5 |
| d1unqa_ | 5.8 | 5.8 | 5.7 | 5.7 | d2fa8a1 | 5.8 | 5.9 | 5.2 | 5.3 |
| d2c60a1 | 6.0 | 6.0 | 6.1 | 6.0 | d1iowa2 | 5.7 | 5.6 | 5.4 | 5.4 |
| d1qwya_ | 5.9 | 5.8 | 5.7 | 5.7 | d1vpla_ | 5.4 | 5.3 | 5.0 | 5.0 |
| d1es9a_ | 5.1 | 5.1 | 4.6 | 4.5 | d2nr4a1 | 5.8 | 5.7 | 5.5 | 5.5 |
| d2opla1 | 5.8 | 5.9 | 5.5 | 5.5 | d1vava_ | 5.6 | 5.5 | 5.4 | 5.4 |
| d1rcqa2 | 5.5 | 5.4 | 4.7 | 4.7 | d2g17a2 | 5.6 | 5.6 | 5.4 | 5.4 |
| d1iqqa_ | 5.6 | 5.6 | 5.4 | 5.3 | d1kxpd2 | 5.7 | 5.6 | 5.4 | 5.3 |
| d1ub3a_ | 5.4 | 5.3 | 4.6 | 4.6 | d1j2ga2 | 5.7 | 5.8 | 5.7 | 5.6 |
| d1o1ya_ | 5.4 | 5.4 | 4.9 | 4.9 | d1puia_ | 5.3 | 5.3 | 4.5 | 4.5 |
| d1zkca1 | 5.9 | 5.8 | 5.7 | 5.7 | d1wb9a3 | 5.6 | 5.6 | 5.2 | 5.3 |
| d2abwa1 | 5.5 | 5.4 | 5.0 | 5.0 | d1uf3a_ | 5.3 | 5.3 | 4.8 | 4.8 |
| d2ez9a1 | 5.5 | 5.5 | 5.0 | 4.9 | d1fxka_ | 6.3 | 6.3 | 5.7 | 5.6 |
| d2ih2a2 | 5.9 | 5.9 | 5.5 | 5.5 | d1vi2a2 | 5.7 | 5.9 | 5.0 | 5.1 |
| d1uzma1 | 5.1 | 5.0 | 4.5 | 4.5 | d2f20a1 | 5.8 | 5.8 | 5.5 | 5.5 |
| d2g82a1 | 5.4 | 5.4 | 4.8 | 4.7 | d2i7ra1 | 6.1 | 6.1 | 5.8 | 5.7 |
| d1uala_ | 5.7 | 5.5 | 5.3 | 5.3 | d1nkqa_ | 5.7 | 5.6 | 5.7 | 5.6 |
| d1fvga_ | 5.5 | 5.5 | 5.3 | 5.2 | d1wrua2 | 6.0 | 5.9 | 5.7 | 5.7 |
| d1vk8a_ | 5.5 | 5.7 | 5.4 | 5.4 | d2d13a1 | 5.5 | 5.5 | 5.0 | 5.0 |
| d1e6ba2 | 6.2 | 6.2 | 5.6 | 5.6 | d1cjxa1 | 6.0 | 6.0 | 5.6 | 5.6 |
| d2vapa1 | 5.1 | 5.1 | 4.6 | 4.6 | d1pv9a2 | 5.3 | 5.3 | 5.3 | 5.2 |
| d1wwza1 | 5.5 | 5.4 | 5.1 | 5.1 | d1zh8a1 | 5.3 | 5.3 | 4.7 | 4.6 |
| d2zcta1 | 5.5 | 5.5 | 5.3 | 5.3 | d2csua3 | 5.2 | 5.3 | 4.6 | 4.6 |
| d1dxja_ | 5.6 | 5.5 | 5.4 | 5.4 | d1gtra1 | 6.1 | 6.0 | 5.7 | 5.6 |
| d1jwqa_ | 5.2 | 5.2 | 4.9 | 4.9 | d1j6ua2 | 5.5 | 5.5 | 4.7 | 4.7 |
| d2nsfa2 | 6.1 | 6.1 | 5.7 | 5.7 | d1u3da2 | 5.5 | 5.5 | 4.9 | 4.8 |
| d3ctka1 | 5.5 | 5.5 | 5.3 | 5.3 | d1m1la_ | 5.5 | 5.5 | 5.4 | 5.4 |
| d1nzna_ | 5.9 | 5.9 | 5.3 | 5.2 | d1wpxb1 | 5.6 | 5.5 | 5.5 | 5.5 |
| d1qo2a_ | 5.4 | 5.4 | 4.7 | 4.7 | d1ovma1 | 5.5 | 5.5 | 5.1 | 5.0 |
| d2dsya1 | 6.0 | 6.2 | 5.8 | 5.8 | d1diha2 | 5.7 | 5.7 | 5.2 | 5.1 |
| d1vbka2 | 5.5 | 5.5 | 5.2 | 5.1 | d2qmwa2 | 5.6 | 5.8 | 5.6 | 5.6 |
| d2c9wc1 | 6.5 | 6.5 | 6.0 | 6.0 | d1j6ra_ | 5.5 | 5.5 | 5.4 | 5.4 |
| d1v77a_ | 5.4 | 5.4 | 4.7 | 4.7 | d2ywqa1 | 5.6 | 5.7 | 5.4 | 5.4 |
| d2f9fa1 | 5.4 | 5.5 | 4.8 | 4.7 | d1egaa2 | 5.6 | 5.6 | 5.3 | 5.2 |
| d1t3ta7 | 5.7 | 5.6 | 5.4 | 5.5 | d1zcca1 | 5.5 | 5.5 | 4.8 | 4.8 |
| d1e4ft1 | 5.7 | 5.6 | 5.4 | 5.4 | d2dbsa1 | 6.2 | 6.4 | 5.6 | 5.9 |
| d1e8ca3 | 5.2 | 5.2 | 4.7 | 4.7 | d1l1ja_ | 5.7 | 5.6 | 5.5 | 5.5 |
| d1xjva2 | 6.0 | 6.0 | 5.8 | 5.7 | d1xata_ | 6.3 | 6.2 | 5.7 | 5.6 |
| d2nzca1 | 5.6 | 5.7 | 5.5 | 5.5 | d1texa_ | 5.4 | 5.4 | 5.1 | 5.0 |
| d2ov9a1 | 5.5 | 5.6 | 5.5 | 5.4 | d3brja1 | 5.8 | 5.8 | 5.4 | 5.4 |
| d1bd0a1 | 5.9 | 5.8 | 5.6 | 5.6 | d2pkgc1 | 6.4 | 6.4 | 5.9 | 5.9 |
| d1gaka_ | 5.9 | 5.9 | 5.5 | 5.5 | d1lnqa2 | 6.1 | 6.1 | 5.6 | 5.6 |
| d1jjta_ | 5.4 | 5.3 | 5.0 | 5.0 | d1snla_ | 6.3 | 6.4 | 5.8 | 5.8 |
| d1n2aa2 | 6.2 | 6.3 | 5.7 | 5.7 | d1x4ga1 | 5.7 | 5.8 | 5.7 | 5.6 |
| d1s3za_ | 5.5 | 5.5 | 5.1 | 5.0 | d2d9ia1 | 5.7 | 5.7 | 5.0 | 5.0 |
| d1ztca1 | 5.6 | 5.5 | 5.1 | 5.1 | d1wgua_ | 5.9 | 5.9 | 5.7 | 5.7 |
| d1fqia_ | 5.9 | 5.9 | 5.4 | 5.3 | d2cr9a1 | 5.8 | 5.9 | 5.7 | 5.6 |
| d1ko3a_ | 5.3 | 5.3 | 5.0 | 5.0 | d1q5fa_ | 5.6 | 5.6 | 5.4 | 5.4 |
| d1t4aa_ | 6.4 | 6.9 | 6.2 | 6.4 | d1dv5a_ | 6.1 | 6.1 | 5.8 | 5.8 |
| d1vqta1 | 5.6 | 5.6 | 4.7 | 4.7 | d1pjza_ | 5.3 | 5.3 | 4.8 | 4.7 |

## Table S2 - Comparison of alignment quality (RMSD_100_) of *ppsAlign* and Fr-TM-align.

The table compares the alignment quality measured in RMSD_100_ of the 100 target proteins using *ppsAlign* and Fr-TM-align.

| SCOP ID | Dataset *D_1_* | | Dataset *D_2_* | | SCOP ID | Dataset *D_1_* | | Dataset *D_2_* | |
| --- | --- | --- | --- | --- | --- | --- | --- | --- | --- |
|  | *ppsAlign* | Fr-TM-align | *ppsAlign* | Fr-TM-align |  | *ppsAlign* | Fr-TM-align | *ppsAlign* | Fr-TM-align |
| d1f9ya_ | 5.2 | 5.2 | 5.0 | 5.0 | d1y6ha_ | 5.7 | 5.6 | 5.4 | 5.4 |
| d1unqa_ | 5.6 | 5.5 | 5.5 | 5.4 | d2fa8a1 | 5.5 | 5.6 | 5.0 | 5.0 |
| d2c60a1 | 5.8 | 5.7 | 5.7 | 5.6 | d1iowa2 | 5.4 | 5.4 | 5.2 | 5.2 |
| d1qwya_ | 5.6 | 5.5 | 5.5 | 5.4 | d1vpla_ | 5.2 | 5.1 | 4.8 | 4.8 |
| d1es9a_ | 4.9 | 4.9 | 4.4 | 4.4 | d2nr4a1 | 5.5 | 5.4 | 5.3 | 5.3 |
| d2opla1 | 5.6 | 5.6 | 5.3 | 5.3 | d1vava_ | 5.4 | 5.3 | 5.2 | 5.2 |
| d1rcqa2 | 5.3 | 5.2 | 4.6 | 4.6 | d2g17a2 | 5.4 | 5.3 | 5.2 | 5.2 |
| d1iqqa_ | 5.4 | 5.3 | 5.2 | 5.2 | d1kxpd2 | 5.5 | 5.4 | 5.2 | 5.1 |
| d1ub3a_ | 5.2 | 5.1 | 4.5 | 4.5 | d1j2ga2 | 5.5 | 5.5 | 5.4 | 5.3 |
| d1o1ya_ | 5.2 | 5.2 | 4.7 | 4.7 | d1puia_ | 5.1 | 5.1 | 4.3 | 4.3 |
| d1zkca1 | 5.6 | 5.6 | 5.5 | 5.4 | d1wb9a3 | 5.3 | 5.3 | 5.0 | 5.0 |
| d2abwa1 | 5.2 | 5.2 | 4.8 | 4.8 | d1uf3a_ | 5.1 | 5.1 | 4.6 | 4.6 |
| d2ez9a1 | 5.3 | 5.3 | 4.8 | 4.7 | d1fxka_ | 6.0 | 5.9 | 5.4 | 5.4 |
| d2ih2a2 | 5.7 | 5.6 | 5.3 | 5.2 | d1vi2a2 | 5.5 | 5.6 | 4.8 | 4.9 |
| d1uzma1 | 4.9 | 4.8 | 4.4 | 4.4 | d2f20a1 | 5.5 | 5.5 | 5.3 | 5.3 |
| d2g82a1 | 5.2 | 5.1 | 4.6 | 4.6 | d2i7ra1 | 5.8 | 5.8 | 5.5 | 5.5 |
| d1uala_ | 5.4 | 5.3 | 5.1 | 5.1 | d1nkqa_ | 5.4 | 5.4 | 5.5 | 5.4 |
| d1fvga_ | 5.2 | 5.2 | 5.0 | 5.0 | d1wrua2 | 5.7 | 5.6 | 5.5 | 5.4 |
| d1vk8a_ | 5.3 | 5.3 | 5.1 | 5.1 | d2d13a1 | 5.3 | 5.2 | 4.8 | 4.8 |
| d1e6ba2 | 5.9 | 5.9 | 5.3 | 5.2 | d1cjxa1 | 5.8 | 5.7 | 5.4 | 5.3 |
| d2vapa1 | 4.9 | 4.9 | 4.4 | 4.4 | d1pv9a2 | 5.1 | 5.1 | 5.1 | 5.1 |
| d1wwza1 | 5.2 | 5.2 | 4.9 | 4.9 | d1zh8a1 | 5.1 | 5.1 | 4.5 | 4.5 |
| d2zcta1 | 5.2 | 5.2 | 5.1 | 5.0 | d2csua3 | 5.0 | 5.1 | 4.4 | 4.4 |
| d1dxja_ | 5.3 | 5.2 | 5.2 | 5.2 | d1gtra1 | 5.8 | 5.7 | 5.4 | 5.4 |
| d1jwqa_ | 5.0 | 5.0 | 4.7 | 4.7 | d1j6ua2 | 5.3 | 5.3 | 4.5 | 4.5 |
| d2nsfa2 | 6.0 | 5.9 | 5.5 | 5.4 | d1u3da2 | 5.3 | 5.3 | 4.7 | 4.7 |
| d3ctka1 | 5.3 | 5.2 | 5.1 | 5.1 | d1m1la_ | 5.3 | 5.3 | 5.2 | 5.2 |
| d1nzna_ | 5.7 | 5.6 | 5.1 | 5.0 | d1wpxb1 | 5.4 | 5.3 | 5.3 | 5.3 |
| d1qo2a_ | 5.2 | 5.2 | 4.5 | 4.6 | d1ovma1 | 5.3 | 5.3 | 4.8 | 4.8 |
| d2dsya1 | 5.7 | 5.7 | 5.5 | 5.4 | d1diha2 | 5.5 | 5.4 | 4.9 | 4.9 |
| d1vbka2 | 5.3 | 5.2 | 5.0 | 5.0 | d2qmwa2 | 5.4 | 5.5 | 5.3 | 5.3 |
| d2c9wc1 | 6.2 | 6.1 | 5.6 | 5.5 | d1j6ra_ | 5.2 | 5.2 | 5.1 | 5.1 |
| d1v77a_ | 5.2 | 5.2 | 4.5 | 4.5 | d2ywqa1 | 5.4 | 5.4 | 5.1 | 5.1 |
| d2f9fa1 | 5.2 | 5.3 | 4.6 | 4.6 | d1egaa2 | 5.3 | 5.3 | 5.0 | 5.0 |
| d1t3ta7 | 5.4 | 5.3 | 5.3 | 5.3 | d1zcca1 | 5.3 | 5.3 | 4.7 | 4.7 |
| d1e4ft1 | 5.4 | 5.3 | 5.2 | 5.1 | d2dbsa1 | 6.0 | 6.0 | 5.4 | 5.4 |
| d1e8ca3 | 5.0 | 5.0 | 4.5 | 4.5 | d1l1ja_ | 5.4 | 5.4 | 5.3 | 5.3 |
| d1xjva2 | 5.8 | 5.7 | 5.5 | 5.4 | d1xata_ | 6.0 | 5.9 | 5.5 | 5.4 |
| d2nzca1 | 5.3 | 5.3 | 5.2 | 5.2 | d1texa_ | 5.2 | 5.2 | 4.9 | 4.9 |
| d2ov9a1 | 5.3 | 5.3 | 5.3 | 5.2 | d3brja1 | 5.5 | 5.5 | 5.2 | 5.2 |
| d1bd0a1 | 5.6 | 5.6 | 5.4 | 5.3 | d2pkgc1 | 6.2 | 6.0 | 5.6 | 5.5 |
| d1gaka_ | 5.6 | 5.5 | 5.3 | 5.2 | d1lnqa2 | 5.9 | 5.7 | 5.4 | 5.2 |
| d1jjta_ | 5.2 | 5.1 | 4.9 | 4.9 | d1snla_ | 6.1 | 5.9 | 5.4 | 5.4 |
| d1n2aa2 | 6.0 | 5.9 | 5.4 | 5.3 | d1x4ga1 | 5.5 | 5.5 | 5.3 | 5.2 |
| d1s3za_ | 5.3 | 5.3 | 4.9 | 4.9 | d2d9ia1 | 5.5 | 5.4 | 4.8 | 4.8 |
| d1ztca1 | 5.4 | 5.3 | 4.9 | 4.9 | d1wgua_ | 5.7 | 5.6 | 5.5 | 5.4 |
| d1fqia_ | 5.6 | 5.6 | 5.1 | 5.1 | d2cr9a1 | 5.6 | 5.6 | 5.4 | 5.4 |
| d1ko3a_ | 5.1 | 5.1 | 4.9 | 4.9 | d1q5fa_ | 5.4 | 5.3 | 5.2 | 5.1 |
| d1t4aa_ | 6.1 | 6.2 | 5.8 | 5.8 | d1dv5a_ | 5.9 | 5.7 | 5.6 | 5.4 |
| d1vqta1 | 5.4 | 5.4 | 4.5 | 4.5 | d1pjza_ | 5.1 | 5.1 | 4.6 | 4.6 |

## Table S3 - Comparison of alignment quality (RMSD_100_) of *ppsAlign* and MAMMOTH.

The table compares the alignment quality measured in RMSD_100_ of the 100 target proteins using *ppsAlign* and MAMMOTH.

| SCOP ID | Dataset *D_1_* | | Dataset *D_2_* | | SCOP ID | Dataset *D_1_* | | Dataset *D_2_* | |
| --- | --- | --- | --- | --- | --- | --- | --- | --- | --- |
|  | *ppsAlign* | MAMMOTH | *ppsAlign* | MAMMOTH |  | *ppsAlign* | MAMMOTH | *ppsAlign* | MAMMOTH |
| d1f9ya_ | 6.1 | 10.0 | 5.8 | 9.4 | d1y6ha_ | 6.6 | 10.9 | 6.1 | 9.9 |
| d1unqa_ | 6.5 | 9.6 | 6.4 | 9.7 | d2fa8a1 | 6.6 | 10.8 | 6.3 | 10.0 |
| d2c60a1 | 6.9 | 10.7 | 7.0 | 10.6 | d1iowa2 | 6.3 | 9.8 | 5.9 | 8.9 |
| d1qwya_ | 6.6 | 13.7 | 6.1 | 11.7 | d1vpla_ | 5.9 | 9.7 | 5.4 | 8.1 |
| d1es9a_ | 5.6 | 8.6 | 5.0 | 7.1 | d2nr4a1 | 6.5 | 10.1 | 6.1 | 9.6 |
| d2opla1 | 6.5 | 11.4 | 6.1 | 10.1 | d1vava_ | 6.2 | 12 | 5.9 | 10.8 |
| d1rcqa2 | 6.0 | 9.3 | 5.1 | 7.1 | d2g17a2 | 6.3 | 10.1 | 6.1 | 9.5 |
| d1iqqa_ | 6.2 | 10.6 | 5.8 | 9.6 | d1kxpd2 | 6.4 | 9.8 | 5.9 | 8.4 |
| d1ub3a_ | 5.8 | 8.4 | 4.9 | 6.1 | d1j2ga2 | 6.5 | 10.7 | 6.4 | 10.2 |
| d1o1ya_ | 6.0 | 9.8 | 5.3 | 7.9 | d1puia_ | 5.8 | 8.5 | 5.0 | 7.2 |
| d1zkca1 | 6.6 | 11.1 | 6.3 | 10.3 | d1wb9a3 | 6.3 | 9.6 | 5.9 | 9.0 |
| d2abwa1 | 6.0 | 10.5 | 5.4 | 8.9 | d1uf3a_ | 5.7 | 8.9 | 5.1 | 6.6 |
| d2ez9a1 | 6.1 | 9.5 | 5.6 | 8.1 | d1fxka_ | 7.4 | 9.9 | 6.5 | 8.8 |
| d2ih2a2 | 6.6 | 12.7 | 6.1 | 11.9 | d1vi2a2 | 6.5 | 10.4 | 5.8 | 9.2 |
| d1uzma1 | 5.6 | 8.5 | 5.0 | 6.6 | d2f20a1 | 6.4 | 11.9 | 6.0 | 10.7 |
| d2g82a1 | 6.0 | 9.4 | 5.4 | 8.2 | d2i7ra1 | 6.8 | 11.2 | 6.4 | 11.1 |
| d1uala_ | 6.3 | 10.1 | 5.9 | 8.5 | d1nkqa_ | 6.3 | 11.5 | 6.2 | 10.6 |
| d1fvga_ | 6.1 | 9.5 | 5.8 | 8.9 | d1wrua2 | 6.7 | 11.2 | 6.3 | 11.0 |
| d1vk8a_ | 6.3 | 9.6 | 6.3 | 9.2 | d2d13a1 | 6.1 | 9.1 | 5.5 | 7.3 |
| d1e6ba2 | 7.0 | 11.5 | 6.6 | 10.3 | d1cjxa1 | 6.7 | 11.0 | 6.3 | 10.9 |
| d2vapa1 | 5.6 | 8.3 | 5.1 | 7.0 | d1pv9a2 | 5.8 | 9.1 | 5.7 | 8.2 |
| d1wwza1 | 6.0 | 9.2 | 5.7 | 8.4 | d1zh8a1 | 5.9 | 9.0 | 5.2 | 7.3 |
| d2zcta1 | 6.1 | 9.4 | 5.8 | 8.4 | d2csua3 | 5.7 | 8.2 | 5.1 | 7.2 |
| d1dxja_ | 6.1 | 10.8 | 5.8 | 9.1 | d1gtra1 | 6.8 | 14.4 | 6.2 | 12.6 |
| d1jwqa_ | 5.8 | 9.1 | 5.4 | 7.9 | d1j6ua2 | 6.1 | 9.2 | 5.3 | 7.7 |
| d2nsfa2 | 6.8 | 11.4 | 6.5 | 10.1 | d1u3da2 | 6.2 | 9.0 | 5.5 | 7.5 |
| d3ctka1 | 6.1 | 9.9 | 5.7 | 8.6 | d1m1la_ | 6.1 | 10.5 | 5.9 | 9.2 |
| d1nzna_ | 6.5 | 9.9 | 5.8 | 9.0 | d1wpxb1 | 6.2 | 11.9 | 6.0 | 10.9 |
| d1qo2a_ | 5.9 | 9.2 | 5.0 | 6.4 | d1ovma1 | 6.1 | 10.0 | 5.6 | 8.7 |
| d2dsya1 | 7.0 | 11.3 | 6.9 | 10.3 | d1diha2 | 6.4 | 10.0 | 6.0 | 9.2 |
| d1vbka2 | 6.1 | 9.3 | 5.6 | 8.3 | d2qmwa2 | 6.4 | 9.7 | 6.6 | 9.3 |
| d2c9wc1 | 7.5 | 11.9 | 7.0 | 10.8 | d1j6ra_ | 6.3 | 9.8 | 6.0 | 8.8 |
| d1v77a_ | 5.9 | 8.8 | 5.0 | 6.7 | d2ywqa1 | 6.3 | 9.6 | 6.2 | 8.8 |
| d2f9fa1 | 6.0 | 9.5 | 5.4 | 8.2 | d1egaa2 | 6.3 | 9.7 | 6.0 | 9.4 |
| d1t3ta7 | 6.4 | 10.0 | 6.0 | 8.8 | d1zcca1 | 6.0 | 9.8 | 5.2 | 7.2 |
| d1e4ft1 | 6.4 | 9.9 | 6.0 | 8.7 | d2dbsa1 | 6.9 | 11.3 | 6.5 | 10.7 |
| d1e8ca3 | 5.8 | 9.1 | 5.1 | 7.2 | d1l1ja_ | 6.2 | 11.4 | 5.9 | 10.5 |
| d1xjva2 | 6.7 | 11.5 | 6.4 | 11.3 | d1xata_ | 6.9 | 13.7 | 6.1 | 11.5 |
| d2nzca1 | 6.4 | 9.4 | 6.6 | 9.1 | d1texa_ | 6.1 | 9.6 | 5.6 | 8.2 |
| d2ov9a1 | 6.1 | 9.3 | 6.0 | 8.9 | d3brja1 | 6.4 | 10.1 | 6.0 | 9.0 |
| d1bd0a1 | 6.6 | 12.6 | 6.2 | 12.3 | d2pkgc1 | 7.3 | 12.5 | 6.9 | 11.2 |
| d1gaka_ | 6.7 | 10.3 | 6.3 | 9.3 | d1lnqa2 | 7.0 | 11.2 | 6.6 | 9.8 |
| d1jjta_ | 5.9 | 9.1 | 5.4 | 7.5 | d1snla_ | 7.3 | 12.7 | 6.6 | 11.3 |
| d1n2aa2 | 7.1 | 11.6 | 6.7 | 10.7 | d1x4ga1 | 6.5 | 10.1 | 6.6 | 10.9 |
| d1s3za_ | 6.1 | 9.4 | 5.7 | 8.6 | d2d9ia1 | 6.6 | 10.4 | 5.9 | 8.9 |
| d1ztca1 | 6.2 | 10.2 | 5.6 | 8.7 | d1wgua_ | 6.7 | 10.2 | 6.5 | 10.4 |
| d1fqia_ | 6.6 | 10.9 | 6.0 | 9.9 | d2cr9a1 | 6.6 | 10.8 | 6.4 | 11.0 |
| d1ko3a_ | 5.8 | 9.3 | 5.4 | 7.7 | d1q5fa_ | 6.2 | 10.3 | 5.9 | 9.6 |
| d1t4aa_ | 7.6 | 13.1 | 7.5 | 12.6 | d1dv5a_ | 6.9 | 12.3 | 6.6 | 11.4 |
| d1vqta1 | 6.1 | 9.3 | 5.0 | 6.7 | d1pjza_ | 5.9 | 9.3 | 5.3 | 7.7 |
